# Surgical treatment of anorectal melanoma: a systematic review and meta-analysis

**DOI:** 10.1093/bjsopen/zrab107

**Published:** 2021-11-30

**Authors:** Esther Jutten, Schelto Kruijff, Anne Brecht Francken, Martijn F Lutke Holzik, Barbara L van Leeuwen, Henderik L van Westreenen, Kevin P Wevers

**Affiliations:** Department of Surgery, Hospital Group Twente, Zilvermeeuw 1, 7609 PP Almelo, the Netherlands; Department of Surgery, University Medical Centre Groningen, Hanzeplein 1, 9713 GZ Groningen, the Netherlands; Department of Surgery, University Medical Centre Groningen, Hanzeplein 1, 9713 GZ Groningen, the Netherlands; Department of Surgery, Isala Zwolle, Dokter van Heesweg 2, 8025 AB Zwolle, the Netherlands; Department of Surgery, Hospital Group Twente, Zilvermeeuw 1, 7609 PP Almelo, the Netherlands; Department of Surgery, University Medical Centre Groningen, Hanzeplein 1, 9713 GZ Groningen, the Netherlands; Department of Surgery, University Medical Centre Groningen, Hanzeplein 1, 9713 GZ Groningen, the Netherlands; Department of Surgery, Isala Zwolle, Dokter van Heesweg 2, 8025 AB Zwolle, the Netherlands; Department of Surgery, Isala Zwolle, Dokter van Heesweg 2, 8025 AB Zwolle, the Netherlands

## Abstract

**Background:**

Anorectal melanoma is a rare neoplasm with a poor prognosis. The surgical approaches for anorectal melanoma can be categorized into local excision (procedures without lymph node removal and preservation of the rectum) and extensive resection (procedures with rectum and pararectal lymph node removal). The aim of this systematic review and meta-analysis was to compare the survival of patients who underwent extensive resection with that of patients who underwent local excision, stratifying patients according to tumour stage.

**Methods:**

A literature review was performed according to PRISMA guidelines by searching MEDLINE/PubMed for manuscripts published until March 2021. Studies comparing survival outcomes in patients with anorectal melanoma who underwent local excision *versus* extensive resection were screened for eligibility. Meta-analysis was performed for overall survival after the different surgical approaches, stratified by tumour stage.

**Results:**

There were 347 studiesidentified of which 34 were included for meta-analysis with a total of 1858 patients. There was no significant difference in overall survival between the surgical approaches in patients per stage (stage I odds ratio 1.30 (95 per cent c.i. 0.62 to 2.72, *P* = 0.49); stage II odds ratio 1.61 (95 per cent c.i. 0.62 to 4.18, *P* = 0.33); stage I–III odds ratio 1.19 (95 per cent c.i. 0.83 to 1.70, *P* = 0.35). Subgroup analyses were conducted for the time intervals (<2000, 2001–2010 and 2011–2021) and for continent of study origin. Subgroup analysis for time interval and continent of origin also showed no statistically significant differences in overall survival.

**Conclusion:**

No significant survival benefit exists for patients with anorectal melanoma treated with local excision or extensive resection, independent of tumour stage.

## Teaser

This systematic review and meta-analysis showed no survival difference between anorectal melanoma patients who underwent extensive resection in comparison with those who underwent local excision, independent of tumour stage. Therefore, local excision should be the primary recommended therapy when technically feasible and adequate local control is expected, improving quality of life in these patients with a short life expectancy.

## Introduction

Anorectal melanoma is a rare neoplasm, with an incidence of 4.8 per 10 million per year^1^. It accounts for only 0.4–1.6 per cent of all malignant melanomas^2^. Patients usually present with non-specific symptoms such as anal pain and mass, a changed defaecation pattern and/or rectal blood loss^2^^,^^3^. This often results in a difficult and delayed diagnostic process. At the time of diagnosis, almost 60 per cent of patients have distant metastases^2^. This subsequently contributes to a poor prognosis of anorectal melanoma, with a 6–22 per cent 5-year survival rate and a median survival of 24 months^2^^,^^4^. Only tumour stage seems to be an independent predictor of survival^5^^,^^6^.

Due to the rare nature of anorectal melanoma, standardized diagnostic and therapeutic international protocols are lacking. The practised surgical approaches for anorectal melanoma can be divided into local excision (procedures without lymph node removal and with preservation of the rectum) and extensive resection (procedures with rectum and pararectal lymph node removal). An extensive resection is a much more invasive procedure with disadvantages such as a longer hospital stay, a longer rehabilitation period and often the burden of a colostomy with negative impact on quality of life^7^. Furthermore, an extensive resection is associated with a higher complication rate, in particular readmission and wound infections but also voiding problems and sexual dysfunction can occur^8–10^.

Local excision is a less invasive procedure and has gained in popularity, as reflected by the increasing adoption of the relatively new transanal minimally invasive surgery (TAMIS) and transanal endoscopic microsurgery (TEM) techniques. Local excision might compromise the chance for adequate local control in some cases^11–13^.

As most patients will have a limited life expectancy, the loss of quality of life after surgery seems highly relevant, especially if less invasive surgical approaches would achieve comparable results for survival rates and local control. Given the low incidence of anorectal melanoma, no prospective studies have been conducted on survival outcomes after surgery, and only retrospective data are available with mostly small sample sizes. Therefore, the aim of this systematic review and meta-analysis was to compare the survival of patients who underwent an extensive resection with that of patients who underwent a local excision, stratified by tumour stage.

## Methods

This systematic review and meta-analysis followed the PRISMA guidelines^14^.

A literature search was performed using MEDLINE/PubMed for all manuscripts published until March 2021. The terms used in this search were ‘Anorectal melanoma’, ‘Anorectal malignant melanoma’, ‘Anal melanoma’, ‘Rectal melanoma’, ‘Surgery’, ‘Surgical’, ‘Treatment’, ‘Excision’, ‘APR’, ‘abdominoperineal resection’, ‘TAMIS’, ‘Transanal Minimally Invasive Surgery’, ‘TME’, ‘Total mesorectal excision’, ‘Rectum amputation’, ‘TEM’, ‘Transanal endoscopic microsurgery’. Cross-references were examined through the database aid ‘similar articles’, and reference lists of the selected articles were scanned for additional potentially relevant studies.

Inclusion criteria were defined according to population, intervention, comparator, outcomes and study design. A publication was considered for inclusion if: the study reported survival data of patients with anorectal melanoma who underwent one of the two different surgical approaches, local excision (local tumour excision, endoscopic resection and TEM) and extensive resection (abdominal perineal resection, total mesorectal excision and rectum amputation); the study reported original data; the outcome measure in terms of 5-year overall survival and/or death events was reported; the study population consisted of a minimum of six patients; and the full-text article was available.

Studies were excluded if they were not written in English. If different studies were published with patients from the same population or with the same source of subject enrolment resulting in data overlap, the most recent study with the largest sample size was included.

In cases of doubt, full-text screening was performed. Each retrieved report was independently evaluated by two investigators for inclusion or exclusion and disagreements were solved by consensus.

### Data extraction

Data extracted from each study included name of primary author, year of publication, country of study origin, study period, mean age, female percentage and survival/death events up to 5-year follow-up after surgery according to tumour stage.

Tumour stage was categorized into the following groups: node-negative disease (stage I), node-positive disease (stage II) and distant metastatic disease (stage III). Node-negative disease was defined as a tumour confined entirely to the anorectum or a tumour infiltrated into the surrounding tissue, without involvement of regional lymph nodes. Node-positive disease was defined as tumour involvement of regional lymph nodes. Distant metastatic disease was defined as metastasis to distant organs or distant lymph nodes^15^. In studies where survival was reported for patients with locoregional stage (stage I and II disease), this was defined as stage I–II. If no distinction was made for stage at all, this was defined as stage I–III.

### Outcomes of interest

The primary outcome of interest was overall survival (defined as the length of time that patients diagnosed with the disease were still alive from the date of diagnosis) of the different surgical approaches, stratified by tumour stage. Also, subgroup analyses were conducted for overall survival of the different surgical approaches for time intervals (up to 2000, 2001–2010 and 2011–2021) and for continent of study origin (North America, Europe and Asia).

### Risk of bias assessment

The risk of bias of the included studies was assessed using the Cochrane Collaboration’s ROBINS-I tool (risk of bias in non-randomized studies and interventions)^16^. Publication bias was examined using funnel plots for outcomes reported by 10 or more studies.

### Statistical analysis

The meta-analysis was performed utilizing Review Manager (RevMan) [Computer program], version 5.3 (Copenhagen: The Nordic Cochrane Centre, The Cochrane Collaboration, 2014).

When continuous data were presented as median and range, means and standard deviations were estimated as previously described^17^. The odds ratio with 95 per cent confidence intervals was calculated for dichotomous variables. The point estimate of the odds ratio value was considered statistically significant at *P* < 0.050 and if the 95 per cent confidence intervals did not cross the value 1.

Heterogeneity between included studies was assessed using the Higgings *I^2^* test. An *I^2^* value greater than 30 per cent was considered to be indicative of substantial heterogeneity. Considering clinical heterogeneity (unknown selection criteria for the surgical approach, risk of bias since no study was randomized) a random-effect model (Mantel–Haenszel) was applied, assuming variations between studies. Funnel plots were constructed to detect the risk of publication bias visually.

## Results

**Fig. 1:**
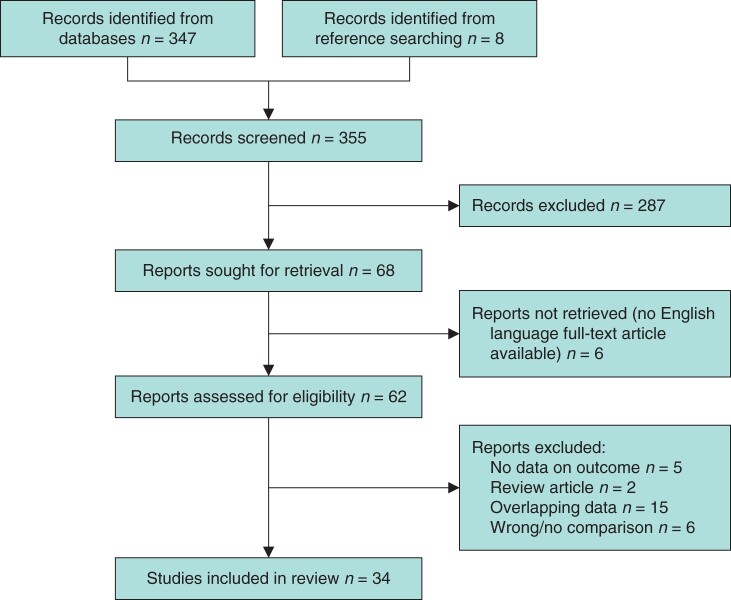
Study selection flow diagram

A total of 347 studies were identified from Medline/Pubmed, however, 287 studies were excluded after screening of titles and/or abstract. Of the remaining 60 publications, six studies^18–23^ were excluded because full text was not written in English. A total of eight records were added through reference searching. This resulted in a total of 62 papers suitable for full text review. Of these, five studies^24–28^ were excluded because there were no data on the outcome measure, two studies^12^^,^^15^ were excluded because they were reviews without original data, 15 studies^29–43^ were excluded due to overlapping data and six studies^44–49^ were excluded because there was no comparison between extensive resection and local excision. Finally, 34 studies comparing survival outcomes after local excision and extensive resection were included for meta-analysis (*Table 1* and *Fig. 1*)^14^.

**Table 1:** Characteristics of included studies

Author, year	Country	Study interval	Mean age (years)	Female (%)	Survival described			Number of patients		
					Per stage	Stage I–II	Stage I–III	Total	ER	LE
Mason and Helwig^50^, 1966	USA	NS	59	24			X	10	7	3
Pack and Martins^51^, 1967	USA	1930–1965	NS	NS	X	X		14	11	3
Wanebo^52^ *et al*., 1981	USA	1950–1977	58	58		X		33	22	11
Cooper^53^ *et al*., 1982	USA	1947–1982	69	68	X	X	X	10	4	6
Siegal^54^ *et al*., 1983	Israel	1960–1981	64	57			X	24	15	9
Angeras^55^ *et al*., 1983	Sweden	1962–1981	65*	64		X		10	6	4
Ward^56^ *et al*., 1986	UK	1938–1982	NS	43		X	X	15	9	6
Kantarovsky^57^ *et al*., 1988	Israel	1960–1980	56	25	X	X		8	2	6
Ross^58^ *et al*., 1990	USA	1952–1988	NS	NS			X	26	14	12
Slingluff and Seigler^59^, 1992	USA	1974–1992	64	71	X			13	6	7
Konstadoulakis^60^ *et al*., 1995	USA	1957–1991	61*	73			X	15	9	6
Thibault^61^ *et al*., 1996	USA	1939–1993	63	70		X		37	26	11
Luna-Perez^62^ *et al*., 1996	Mexico	1980–1996	66	54	X		X	15	12	3
Weyandt^63^ *et al*., 2003	Germany	1992–2001	62	47			X	13	5	8
Bullard^10^ *et al*., 2003	USA	1998–2002	65	56		X		15	4	11
Moozar^64^ *et al*., 2003	Canada	1980–1999	56	64			X	14	4	10
Malik^65^ *et al*., 2004	USA	1983–2001	61	47			X	18	7	11
Pessaux^66^ *et al*., 2004	France	1977–2002	58	70		X		30	9	21
Ishizone^67^ *et al*., 2008	Japan	1997–2006	66	57			X	57	47	10
Belli^68^ *et al*., 2009	Italy	1975–2006	62*	52		X		31	13	18
Nilsson and Ragnarsson-Olding^69^, 2010	Sweden	1960–1999	69*	60			X	152	66	86
Zhang^70^ *et al*., 2010	China	1995–2007	53	61		X		54	39	15
Aytac^71^ *et al*., 2010	Turkey	1997–2004	58	57			X	14	11	3
Choi^72^ *et al*., 2011	Korea	1999–2008	62*	58			X	19	12	7
Che^73^ *et al*., 2011	China	1975–2008	55	61			X	56	36	20
Wang^74^ *et al*., 2013	China	1989–2011	54*	65		X		43	37	6
Yen^75^ *et al*., 2013	Taiwan	1993–2011	58	64			X	21	13	8
Perez^76^ *et al*., 2013	USA	1985–2010	61*	52			X	65	25	40
Miguel^77^ *et al*., 2015	Portugal	2000–2011	63*	80		X		6	5	1
Chen^4^ *et al*., 2016	China	1973–2011	68	63	X	X	X	317	105	212
Nusrath^5^ *et al*., 2018	India	2010–2015	NS	50	X	X		20	15	5
Kaya^78^ *et al*., 2018	Turkey	2010–2017	69	80			X	10	5	5
Ford^79^ *et al*., 2018	USA	2004–2014	68*	59		X		570	383	187
Jutten^6^ *et al*., 2021	Netherlands	1989–2019	67	60	X	X		103	44	59
Mean(s.d.)			62(4.7)	58(12.7)						
Total					8	17	19	1858	1028	830

*Method of Hozo *et al*.^17^ applied to estimate respective means. ER, extensive resection; LE, local excision; NS, not stated.

### Patients and study characteristics

All 34 included papers were retrospective data reports. The reports were published between 1966 and 2021, and 35 per cent of the studies were conducted in the USA. The mean age of patients was 62 (range 53–69) years. The mean percentage of female patients reported in studies was 58 per cent. Eight studies reported survival outcome per stage, 17 studies reported survival in patients with locoregional disease and 19 studies reported survival without making distinction in stage of disease. Altogether 1858 patients were involved, of these 1028 patients underwent extensive resection and 830 underwent local excision.

### Survival outcomes

**Fig. 2:**
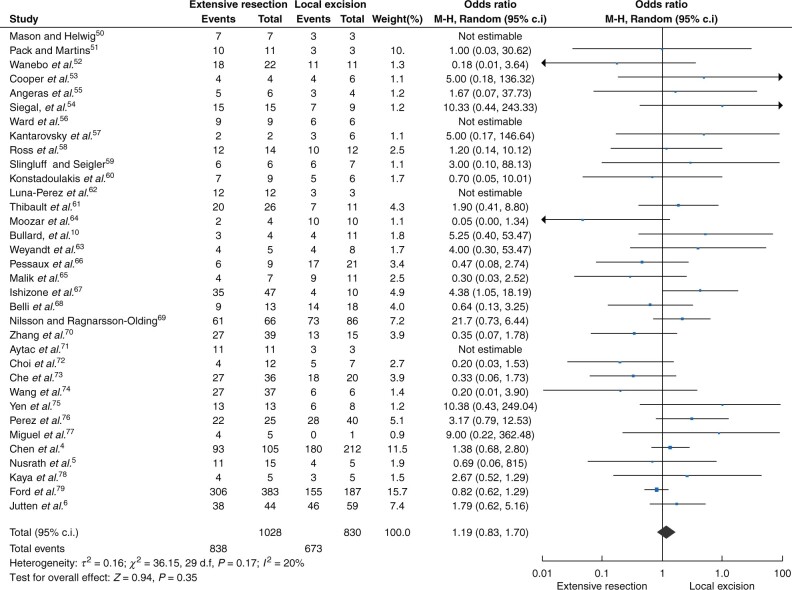
Forest plot of the overall survival of the different surgical approaches in all patients without stage stratification (stage I–III) M-H, Mantel–Haenszel

**Fig. 3:**
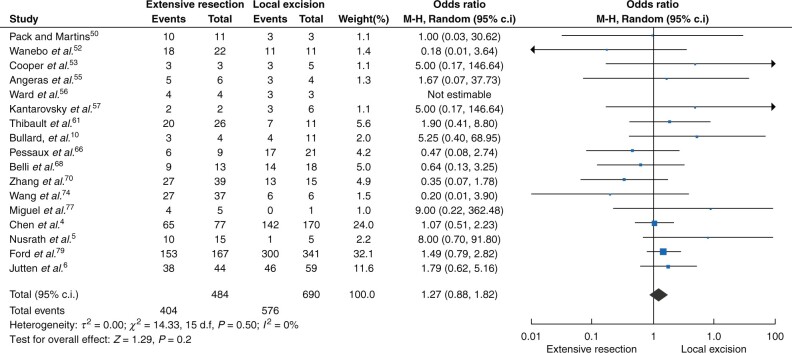
Forest plot of the overall survival of the different surgical approaches in patients with stage I–II disease M-H, Mantel–Haenszel

**Fig. 4:**
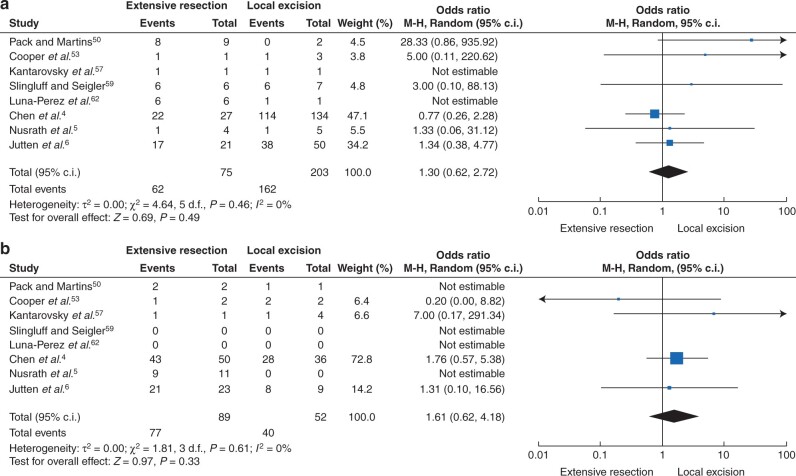
Forest plot of the overall survival of the different surgical approaches **a** In patients with stage I disease. **b** In patients with stage II disease. M-H, Mantel–Haenszel

There was no significant difference in overall survival between the different surgical approaches in all patients without stage stratification (stage I–III, 1858 patients, odds ratio 1.19 (95 per cent c.i. 0.83 to 1.70, *P* = 0.35)) and there was no between-study heterogeneity observed (*I^2^* = 20 per cent, *P* = 0.17) (*Fig. 2*). Likewise, for patients with locoregional disease (stage I–II, 1174 patients) extensive resection and local excision showed equivalent results in terms of survival (odds ratio 1.27 (95 per cent c.i. 0.88 to 1.82, *P* = 0.20); *I^2^* = 0 per cent, *P* = 0.50)) (*Fig. 3*). For patients with stage I disease (*Fig. 4a*, 278 patients) and stage II disease (*Fig. 4b*, 127 patients), no significant improvement of survival was shown for either of the surgical approaches (stage I disease, odds ratio 1.30 (95 per cent c.i. 0.62 to 2.72, *P* = 0.49) (*Fig. 4a*); stage II disease, odds ratio 1.61 (95 per cent c.i. 0.62 to 4.18, *P* = 0.33) (*Fig. 4b*)). In both analyses, no significance in between-study heterogeneity was observed.

### Subgroup analysis

Subgroup analyses were conducted to assess consistency of conclusions over the years and between different continents of origin (*Table 2*). There were no statistically significant differences in overall survival between patients who underwent extensive resection in comparison with that of patients who underwent local excision regardless of time interval or continent of origin.

**Table 2:** Subgroup analysis for overall survival of the different surgical approaches for time intervals and continent of origin

	No of studies	No of participants	Odds ratio	***P***	*I^2^* (%)
**Time interval**					
Up to 2000	13	230	1.67 (0.73, 3.83)	0.23	0
2001–2010	10	398	1.02 (0.44, 2.40)	0.96	48
2011–2021	11	1230	1.11 (0.67, 1.85)	0.68	31
**Continent**					
North America	14	855	1.06 (0.61, 1.83)	0.84	13
Europe	8	360	1.53 (0.84, 2.80)	0.16	0
Asia	12	643	1.11 (0.51, 2.40)	0.79	41

Values in parentheses are 95 per cent confidence intervals. Odds ratio >1 favours local excision.

### Risk of bias across studies

The risk of bias of the selected studies is shown in *Table S1*, and no study was classified as ‘critical’. Outcomes reported by at least 10 studies (overall survival of the different surgical approaches in all patients without stage stratification and overall survival of the different surgical approaches in patients with stage I–II disease) were examined for publication bias using funnel plots (*Fig*. *S1*). In both plots, a symmetrical inverted funnel shape is seen, suggesting that publication bias was unlikely.

## Discussion

This systematic review and meta-analysis documented that survival outcomes of anorectal melanoma patients are not different when treated with local excision or extensive resection. This finding was not affected by tumour stage, regardless of time interval and continent of study origin.

Two previous systematic reviews with meta-analysis were conducted in this field^12^^,^^13^. The first one included 31 studies with a total of 1006 patients from 1966–2013^12^. The authors concluded that overall survival did not differ significantly between the extensive resection (in their study abdominoperineal resection) and local excision groups with an odds ratio of 1.14 (95 per cent c.i. 0.74 to 1.76, *P* = 0.54) without significant between-study heterogeneity (*I^2^* = 21 per cent, *P* = 0.17), but they also concluded that an abdominoperineal resection might confer better local control. The latter study included 23 studies (1990–2016) with a total of 895 patients^13^. The results in that systematic review also demonstrated no significant difference in overall survival between the surgical strategies, however the authors did not find a significant improvement in local control with the use of extensive resection (abdominoperineal resection) over local excision. Both previous systematic reviews made no distinction between tumour stage, which is an independent factor for survival^5^^,^^6^, studies with overlapping data were not excluded, and no subgroup analyses were performed to assess consistency of conclusions over the years and between different continents of origin. Since the publication of these manuscripts, additional studies have been conducted and transanal surgical approaches like TAMIS and TEM are more widely used. Moreover, diagnostic techniques and adjuvant treatment strategies have changed extensively over the last decade. This implementation resulted in almost double the number of patients included in the present meta-analysis.

The present study demonstrated that, independent of tumour stage, there is no significantly better survival rate for one of the surgical approaches regardless of time interval and continent of study origin. Although in Asian countries it is more common practice to perform an extended lymph node dissection^80^^,^^81^, the present study did not find a better survival rate in Asian countries. This is in line with previous studies^61^^,^^76^, which conclude that (inguinal and mesorectal) lymphadenectomy in anorectal melanoma patients does not ameliorate the prognosis in case of nodal metastasis. In particular, one of these studies suggested anorectal melanoma may skip lymphatic spread and metastasize haematogenously to distant sites^76^. Over time, newer systemic treatment modalities have been added to the surgical therapy, but this has not resulted in a survival benefit for one of the surgical approaches. Moreover, survival has not improved over the past three decades.

Revealing that survival of patients with anorectal melanoma has not improved at all during the last three decades indicates the need for personalized treatment, focusing on local control and quality of life, preferably in a multidisciplinary setting. In addition, it suggests the need for newer treatment modalities like immunotherapy and targeted therapies. Although cutaneous melanomas are found to be highly immunogenic, this has not been shown yet for anorectal or other mucosal melanomas^82^. This suggests mucosal melanomas might have a different aetiology and that further investigations on this subject are necessary.

The main limitation of this meta-analysis is the retrospective design of all included studies. Due to the rare nature of this disease, no randomized controlled trials are available or will be available in the future. However, this may have led to a selection bias for choosing the surgical procedure. Also, data are lacking on whether resection margins were microscopically negative (R0) and local recurrence, which might influence survival. Furthermore, there must have been variations in (neo)adjuvant treatments among the included studies and the surgical procedures have evolved over time, which have not been taken into account in this meta-analysis other than that the authors looked at differences for subsequent time intervals and geographical locations of treatment. Still, this systematic review and meta-analysis represents a large collective of data on anorectal melanomas and investigates survival stratified by tumour stage.

Since there is no clear survival benefit for extensive resection compared with local excision, local surgical control and quality of life merit consideration in patients with a short life expectancy. The local recurrence rate seems similar for wide local excision (37 per cent) and abdominoperineal resection (34 per cent)^13^. Extensive resection results in worse quality of life in comparison with local excision^83^^,^^84^. This applies in particular to functional outcome, body image and urological problems. Also, patients who undergo extensive resection are more likely to experience sexual problems^83^^,^^84^. Likewise, the time of recovery after extensive resection will take longer in comparison with local excision. The recovery period until full fitness is longer for patients who undergo extensive resection, whereas patients who undergo a local excision procedure are expected to have a quick recovery with early resumption of normal activities^85^^,^^86^. In patients with a short life expectancy, this could be a very valuable time. However, patient symptoms, tumour sphincter invasion or technical feasibility can be reasons for extensive resections.

## Supplementary Material

zrab107_Supplementary_Data
