# Supplementary material for: Surgical treatment of anorectal melanoma: a systematic review and meta-analysis
Source: BJS Open. 2021 Nov 30;5(6):zrab107. doi: 10.1093/bjsopen/zrab107 (PMC8675246; doi:10.1093/bjsopen/zrab107)
Supplement: zrab107_Supplementary_Data [file zrab107_supplementary_data.zip › Supplementary_Table.docx]

| **Supplementary Table 1. Risk of bias of individual studies based on ROBINS-I tool** | | | | | | | | |
| --- | --- | --- | --- | --- | --- | --- | --- | --- |
| **Author** | **Bias due to confound-ding** | **Bias in selection of partici-pants into the study** | **Bias in classify-cation of interventions** | **Bias due to deviations from intended inter-ventions** | **Bias due to missing data** | **Bias in measure-ment of outcomes** | **Bias in selection of the reported result** | **Overall assess-ment** |
| Mason^59^ | Moderate | Low | Low | Low | Moderate | Low | Low | Moderate |
| Pack^60^ | Moderate | Moderate | Low | Low | Moderate | Low | Low | Moderate |
| Wanebo^61^ | Moderate | Moderate | Low | Low | Moderate | Low | Low | Moderate |
| Cooper^62^ | Serious | Moderate | Moderate | Low | Low | Low | Low | Serious |
| Siegal^63^ | Moderate | Low | Low | Moderate | Low | Low | Low | Moderate |
| Angeras^64^ | Serious | Low | Low | Moderate | Low | Low | Low | Serious |
| Ward^65^ | Moderate | Low | Moderate | Low | Low | Low | Low | Moderate |
| Kantarovsky^66^ | Serious | Low | Moderate | Low | Low | Low | Low | Serious |
| Ross^67^ | Moderate | Low | Low | Low | Low | Low | Low | Moderate |
| Slingluff^68^ | Serious | Low | Moderate | Moderate | Low | Low | Low | Serious |
| Konstadou-lakis^69^ | Moderate | Low | Low | Low | Low | Low | Low | Moderate |
| Thibault^52^ | Moderate | Low | Low | Low | Low | Low | Low | Moderate |
| Luna-Perez^70^ | Serious | Low | Low | Low | Low | Low | Low | Serious |
| Weyandt^71^ | Serious | Low | Low | Low | Low | Low | Low | Serious |
| Bullard^10^ | Moderate | Low | Low | Low | Low | Low | Low | Moderate |
| Moozar^72^ | Serious | Moderate | Low | Moderate | Moderate | Low | Low | Serious |
| Malik^73^ | Moderate | Low | Low | Low | Low | Low | Low | Moderate |
| Pessaux^74^ | Moderate | Low | Low | Moderate | Low | Low | Low | Moderate |
| Ishizone^75^ | Low | Serious | Low | Moderate | Low | Low | Low | Serious |
| Belli^76^ | Low | Low | Low | Moderate | Moderate | Low | Low | Moderate |
| Nilsson^77^ | Low | Low | Low | Moderate | Low | Low | Low | Moderate |
| Zhang^78^ | Low | Low | Low | Low | Moderate | Low | Low | Moderate |
| Aytac^79^ | Serious | Low | Low | Serious | Low | Low | Low | Serious |
| Choi^80^ | Moderate | Serious | Low | Moderate | Low | Low | Low | Serious |
| Che^81^ | Low | Moderate | Low | Moderate | Low | Low | Low | Moderate |
| Wang^82^ | Moderate | Low | Low | Moderate | Low | Low | Low | Moderate |
| Yen^83^ | Moderate | Low | Low | Low | Moderate | Low | Low | Moderate |
| Perez^53^ | Low | Low | Low | Moderate | Low | Low | Low | Moderate |
| Miguel^84^ | Serious | Low | Low | Moderate | Low | Low | Low | Serious |
| Chen^4^ | Low | Low | Low | Low | Moderate | Low | Low | Moderate |
| Nusrath^5^ | Moderate | Low | Low | Low | Moderate | Low | Low | Moderate |
| Kaya^85^ | Serious | Low | Low | Moderate | Low | Low | Low | Serious |
| Ford^86^ | Low | Low | Low | Moderate | Low | Low | Low | Moderate |
| Jutten^6^ | Low | Low | Low | Low | Moderate | Low | Low | Moderate |
|  |  |  |  |  |  |  |  |  |
