# Supplementary figures and images for: Surgical treatment of anorectal melanoma: a systematic review and meta-analysis
Source: BJS Open. 2021 Nov 30;5(6):zrab107. doi: 10.1093/bjsopen/zrab107 (PMC8675246; doi:10.1093/bjsopen/zrab107)

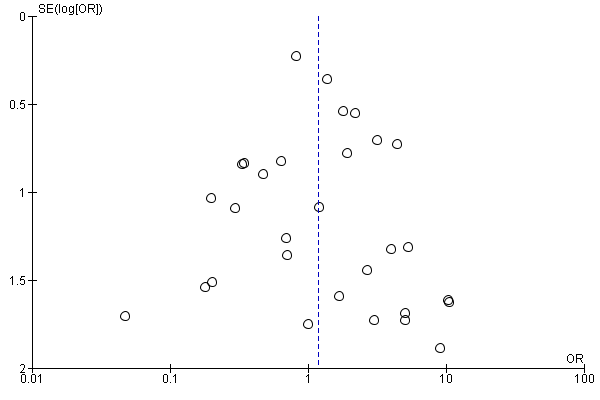

Supplement: zrab107_Supplementary_Data [file zrab107_supplementary_data.zip › Supplementary_Figure_1A.png]

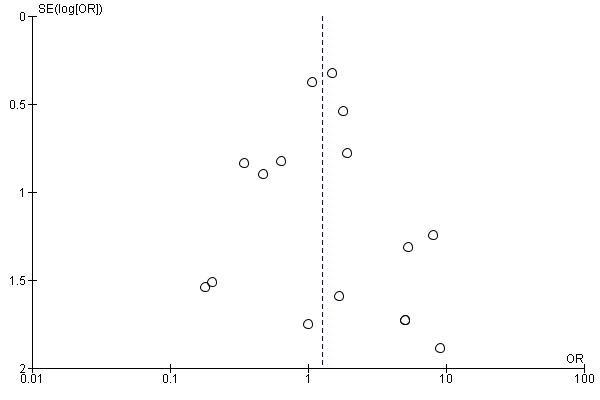

Supplement: zrab107_Supplementary_Data [file zrab107_supplementary_data.zip › Supplementary_Figure_1B.png]
